# Supplementary material for: Increased Number of Mucosal-Associated Invariant T Cells Is Associated with the Inhibition of Nonalcoholic Fatty Liver Disease in High Fat Diet–Fed Mice
Source: Int J Mol Sci. 2022 Dec 4;23(23):15309. doi: 10.3390/ijms232315309 (PMC9739562; doi:10.3390/ijms232315309)
Supplement: Supplementary file 1 [file ijms-23-15309-s001.zip › ijms-2030289-supplementary.pdf]

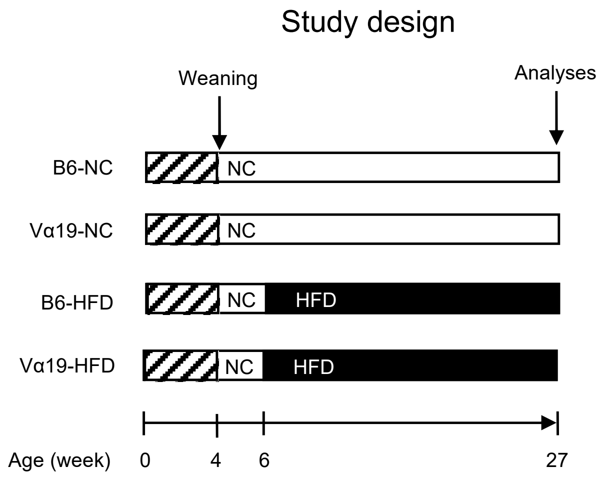

Supplementary Figure S1.

Study design. Mice were assigned to four groups: C57BL/6NJcl mice on a normal chow diet (B6-NC; n=9~18), Vα19 mice on normal chow diet (Vα19-NC; n=9~18), C57BL/6 mice fed on a high-fat diet (B6-HFD; n=9~18), and Vα19 mice fed on a high-fat diet (Vα19-HFD; n=9~18). The mice in NC groups were fed on a normal chow diet after weaning at 4 weeks to 27 weeks of age. Mice in HFD groups were fed on a normal chow diet after weaning to 6 weeks and then on a high-fat diet from 6 weeks to 27 weeks of age. The most analyses were performed at 27 weeks of age.

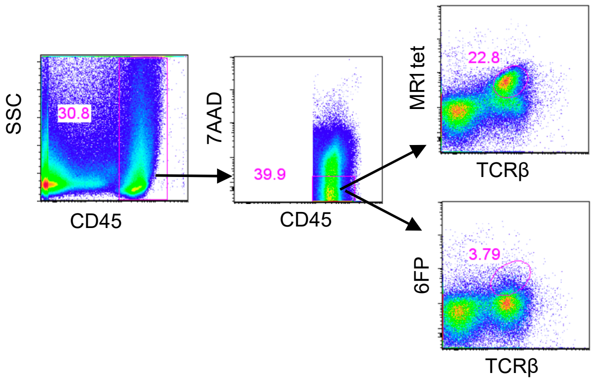

Supplementary Figure S 2.

Identification of MAIT cells in the liver of normal chow-fed Vα19 mice by flowcytometry. MAIT cells were defined as both TCRβ- and MR1 tetramer-positive but 6FP-negative cells in the CD45-positive but 7AAD-negative fraction. A representative result is shown.

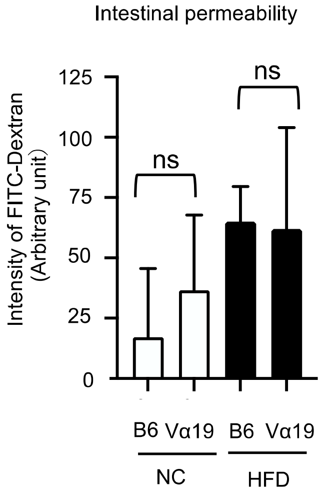

Supplementary Figure S3

Intestinal permeability was evaluated by the leakage of FITC-dextran from intestine to circulating blood as described in Materials and Methods. Serum FITC levels were measured in C57BL/6 mice (B6) and Vα19 mice (Vα19) fed on normal chow diet (NC) and high-fat diet (HFD) at 27 weeks of age. n= 6 in each group. ns; statistically not significant.
